# Variability of Performance and Kinematics of Different Shot Put Techniques in Elite and Sub-Elite Athletes–A Preliminary Study

**DOI:** 10.3390/ijerph19031751

**Published:** 2022-02-03

**Authors:** Andrzej Mastalerz, Jerzy Sadowski

**Affiliations:** 1 Department of Biomedical Sciences, Faculty of Physical Education, Józef Piłsudski University of Physical Education in Warsaw, 00-968 Warsaw, Poland; 2 Department of Sport Sciences, Faculty of Physical Education and Health, Józef Piłsudski University of Physical Education in Warsaw, 00-968 Warsaw, Poland; jerzy.sadowski@awf.edu.pl

**Keywords:** throwing, relative error, biomechanical parameters, 3D kinematic analysis

## Abstract

The purpose of the study was to analyze the variability of performance and kinematics of different shot put techniques in elite athletes (group A: 5 athletes using the rotational technique and 3 athletes using the glide technique) and sub-elite athletes (group B: 3 athletes using the rotational technique and 3 athletes using the glide technique). Each athlete performed 6 trials. Only 34 trials in group A and 27 trials in group B were analyzed. Two high-speed digital cameras were positioned 8 m from the center of the shot put throwing circle. All throws performed during international and national competitions were analyzed using the Ariel Performance Analysis System. To estimate variability of kinematic parameters, the value of relative error was calculated. The average relative error generally showed low variability for the analyzed indicators. In only 4 analyzed cases, variability was high (>20%). Statistical analysis was used to find indicators which have a significant influence on the distance of the throw (according to the sports level and technique). Significant inverse correlations (at *p* < 0.05) between the distance and the average relative error of the selected indicators were mainly obtained, which meant that the distance was longer when the value of variability (average relative error) was low. The research results show that greater repeatability of the technique (lower RE) has a significant impact on the length of the shot put.

## 1. Introduction

Several throwing techniques of the shot put (glide, rotational, leg reverse, shuffle) have been trialed over the years. The gliding technique was introduced in the 1950s and was prevalent for many years. The gliding technique involves a linear push out of the back to the front of the circle while facing away from the sector. Then, shot putters rotate their body toward the throwing section, putting the shot for maximum distance. The rotational style appeared in the 1970s and is the most popular nowadays [1]. The rotational technique is traditionally separated into six phases and involves approximately two rotations of the body before implementing the release, during which an athlete generates large forces within short time frames to project the shot into the sector. Large forces result from both technique-related and physical factors during the shot put, and consideration of both these types of factors is needed to enhance performance. In particular, the rotational shot put technique as a complex movement requires a high level of motor control [2]. The initial movement in the shot put techniques is generated by the power of the lower body segment muscles (legs), while the final movement is generated by the power of the upper body segment muscles (arm-hand) [3]. The thrower must generate speed, multiply it by using the chain of power in their body and transfer it into the throw. The parameters of technique and power in the shot put are closely related. The amount of shot put force mainly depends on the power, while distance and/or time during which the force is applied to the shot depends on the technique. Height of release, angle of release and release velocity have been the technical parameters analyzed in the studies on throwing events such as the shot put. Among these parameters, release velocity is the most important as the horizontal displacement of the shot is proportional to velocity squared [4]. The correlation between release velocity and the best results is strong. Release velocity is generated by preceding phases, especially the second double support phase [3]. Release velocity is also inversely proportional to the angle of release [5,6]. Release speed decreases with an increase in the angle, approximately 1.7 (m/s)/rad and decreases with an increase in height, approximately 0.8 (m/s)/m. The angle of release is the second most important factor in the projectile motion equation. It is determined by the angle of the throwing arm and the orientation of the trunk relative to the ground. According to McCoy et al. [7] and Hay [8], the height of release is mainly determined by the athlete’s anthropometric parameters (the height and the length/span of the throwing arm). In addition to the release parameters, scientists have determined parameters of the body which are most critical for success in the shot put. Alexander et al. [9] examined 31 male and 30 female shot putters and noticed that for the female throwers, the most critical parameters to produce longer distance were knee extension during the glide, elbow speed during delivery, and a greater shoulder flexion angle at release. As for the male throwers, the most significant parameters included center of mass speed during the glide, vertical acceleration of the center of mass during the delivery, and a trunk angle at the start of the glide. 

The use of 3D video motion analysis helped athletes and their coaches to analyze the technique they use (rotational or glide technique) and to find ways to increase efficiency and improve performance.

Kinematics of motion is not the same, even during repeated execution of the same movement. Natural variations in the position, velocity and acceleration of the body limbs take place even during apparently identical movements and influence the variability of the throw kinematics [10]. Consequently, both coaches and biomechanists quantify kinematics and kinetics when working with a shot putter to enhance performance [11,12,13,14,15,16]. Therefore, the aim of the study was to measure variability of selected kinematic parameters (using average relative error) in relation to the athletes’ sports levels (international level and national level) and technique (glide and rotational technique). Another aim was to assess the correlation between average relative errors of selected biomechanical indicators and the distance of the throw.

## 2. Material and Methods

Fourteen elite and sub-elite shot putters (right-handed) took part in this analysis. Eight elite participants, including 5 athletes using the rotational technique and 3 athletes using the glide technique, performed trials during an international competition (group A), whereas six sub-elite participants (3 athletes using the rotational technique and 3 athletes using the glide technique) performed trials during national championships (group B). In the year of the analyzed competition, all athletes from group A belonged to the group of thirty world-leading shot putters (places from 18th to 37th), with the best results in the range between 20.38 m and 20.64 m. National ranking of 100 best results of the year in the shot put included performances of 6 competitors from group B. The best results of the athletes taking part in the analyzed national competition (group B) ranged from 20.32 m to 18.77 m (places from 11th to 67th for the best trials of the year). The shot putters from group A who used the glide technique (age: 26.3 ± 3.2 y; body mass: 124 ± 6.9 kg; body height: 1.95 ± 0.08 m; BMI: 32.5 ± 0.8 kg/m^2^) were slightly taller (3 cm on average), had greater weight and BMI (7.2 kg and 0.6 kg/m^2^) and were a little older (1.3 years) than shot putters who used the rotational technique in the same group (age: 25 ± 1 y; body mass: 116.8 ± 8.6 kg; body height: 1.92 ± 0.07 m; BMI: 31.9 ± 1.1 kg/m^2^). A similar dependence (with slightly greater differences between the two techniques) was observed in athletes in group B. Athletes who used the glide technique (age: 25.7 ± 2.5 y; body mass: 122.7 ± 6.1 kg; body height: 1.92 ± 0.06 m; BMI: 33.3 ± 1.6 kg/m^2^), were 3.7 years older, 3 cm taller, 12.7 kg heavier and had BMI about 2.4 kg/m^2^ greater than athletes who used the rotational technique (age: 22 ± 2 y; body mass: 110 ± 7.1 kg; body height: 1.89 ± 0.02 m; BMI: 30.9 ± 1.3 kg/m^2^). There were no significant differences (*p* < 0.05) for the height of the athletes’ center of gravity depending on the group and technique (F(1,7) = 0.7484, *p* = 0.416).

Each athlete performed 6 trials during a competition in the final session. Only the measured trials (34 in group A and 27 in group B) were analyzed. In group A, 5 athletes who used the rotational technique performed 21 measured trials, while 3 athletes who used the glide technique performed 13 measured trials. In group B, 3 athletes who used the rotational technique performed 13 measured trials, while 3 athletes who used the glide technique performed 14 measured trials. All participants gave their informed consent and were informed of the benefits and risks of the investigation prior to signing an institutionally approved informed consent document to take part in the study. The study was conducted in accordance with the Declaration of Helsinki, and the protocol was approved by the Ethics Committee of Józef Piłsudski University of Physical Education in Warsaw (SKE 01–02/2013).

Two high-speed digital cameras (JVC, GR DVL-9800, Victor Company of Japan, Yokohama, Japan) were positioned perpendicular to each other and 8 m from the center of the shot put throwing circle.

All throws performed during international and national competitions were recorded and then analyzed using the Ariel Performance Analysis System (APAS). Eighteen points were digitized. Sixteen points were placed on the athlete’s body, including the big toe, ankle, knee, hip, wrist, elbow, and shoulder for the left and right side of the body as well as the right hand, the chin and the top of the head. The 17th point was placed in the center of the shot, and the last (18th) was placed on the right-side edge of the toe-board of the throwing circle. The analyzed area of the throwing circle was calibrated with a 1.5 m × 2 m × 1.5 m reference scaling frame. The calibration was performed before and after the competition session. Synchronized data sequences from all camera views were utilized.

The kinematic parameters of the athlete and the shot during release (RLS–the last contact of the athlete) presented in Table 1 were taken into account during the analysis that included the measured trials only. 

To estimate variability of kinematic parameters, the value of relative error (RE) was calculated.

### Statistical Analysis

Two-factor analysis of variance (ANOVA) was used to analyze significant differences depending on the two factors: the GROUP (sports level) and/or the TECHNIQUE (rotational/glide technique).

Correlations between selected indicators were determined using Pearson’s correlation coefficient. All statistical analyses were based on RE of selected indicators. The use of RE made it possible to perform a statistical analysis of all shot-put measured trials. The values of the distance of the throws were used in the Pearson’s correlation analysis with RE of the selected indicators to find the most important correlations between them. Statistica 10.0 (StatSoft, Inc., (2011) STATISTICA (Data Analysis Software System), Version 10. http://www.statsoft.com (accessed on 1 December 2019)) was used for statistical analysis. The significance level α = 0.05 was used to assess the significance of differences and correlations.

## 3. Results

The angle indicators (Table 2) like β_p, λ_l, z_CG, R_CG, z_S, R_S, differed more in the rotational technique than in the glide one. The most similar values (without the division into the groups and techniques) were noted for the height (h) and the distance in the horizontal direction of the athlete’s center of gravity movement (x_CG). Right hip angle (λ_p), horizontal velocity of the athlete’s center of gravity (Vx CG), the height of the shot (H) and the angle of release (γ) differed more in the glide technique.

The greatest differences were found for the S-H as the effect of GROUP and TECHNIQUE. Other indicators of the athlete and the shot had low RE value (Table 3).

Statistically significant influence of the GROUP factor (elite, sub-elite) was noted for mean RE: δ_r (F(1.57) = 35.432, *p* ≤ 0.0001), λ_r (F(1.57) = 14.890, *p* ≤ 0.001) φ_r (F(1.57) = 16.306, *p* = 0.002), S-H (F(1.57) = 9.545, *p* = 0.003), γ (F(1.57) = 9.552, *p* = 0.003), VxCG (F(1.57) = 7.547, *p* = 0.008), VxS, VyS, VrS (F(1.57) = 10.657, *p* = 0.002, F(1.57) = 16.897, *p* ≤ 0.001, F(1.57) = 28.846, *p* ≤ 0.001, respectively) and also for h (F(1.57) = 13.0, *p* ≤ 0.001). Statistically significant influence of the TECHNIQUE was observed for mean RE of the 3 indicators: φ_r (F(1.57) = 5.461, *p* = 0.002), S-H (F(1.57) = 10.462, *p* = 0.002), γ (F(1.57) = 6.950, *p* = 0.011) and for RE of VxS (F(1.57) = 6.052, *p* = 0.017). Both GROUP and the TECHNIQUE factors significantly influenced mean RE of φ_r (F(1.57) = 18.377, *p* ≤ 0.001), δ_r (F(1.57) = 4.574, *p* = 0.037), VyS (F(1.57) = 4.766, *p* = 0.332), γ (F(1.57) = 4.887, *p* = 0.031), and D (F(1.57) = 14.417, *p* ≤ 0.001).

The highest level of RE (about 20%) was found for the horizontal velocity of CG in both groups and techniques and for the vertical velocity of CG except for the rotational technique in group A (Table 4). Levels of RE of the shot velocities were definitely lower than the velocities of the athlete’s center of gravity. Generally lower levels of RE for the athlete’s center of gravity than for the shot were observed for the horizontal and resultant path of CG.

Depending on the GROUP factor (elite, sub-elite) only, statistically significant differences were noted for RE level of R_S (F(1.57) = 8.107, *p* = 0.006) and z_S (F(1.57) = 6.042, *p* = 0.017). In the case of the technique (without the division into sports levels), significant differences were found for RE of x_CG, y_CG, z_CG and R_S (F(1.57) = 5.795, *p* = 0.019, F(1.57) = 11.670, *p* = 0.001, F(1.57) = 17.312, *p* ≤ 0.001, F(1.57) = 9.464, *p* = 0.003, respectively) and also for RE of z_S (F(1.57) = 16.414, *p* ≤ 0.001). Both the GROUP and the TECHNIQUE factors significantly influenced RE level of x_CG (F(1.57) = 4.982, *p* = 0.030), y_CG ((F(1.57) = 4.982, *p* = 0.030), and R_S, (F(1.57) = 8.205, *p* = 0.006).

All the statistically significant correlations between the distance of the throw and RE of selected release indicators had inverse correlations, which meant that the lower the variability of the indicator, the longer the distance was (Table 5). The inverse correlations between the distance and RE of the angle of the right and left knee were observed especially in the rotational technique in group B. RE of the vertical and horizontal velocity of CG exerted a significant influence on the throw distance, but only in the glide technique in group A. A significant influence of the resultant velocity of the shot and of release angle on the distance was confirmed. In four cases, there were significant inverse correlations between the distance and RE of the resultant velocity of S. Also, in three cases, significant inverse correlations between the distance and RE of the vertical velocity of the shot were noted. 

## 4. Discussion

The most important observation of the study was the identification of variability in performance and kinematics for elite and sub-elite shot putters. This study fills gaps that have not been previously addressed in research on the shot put. Release indicators have been frequently examined in the competition like the shot put [4,5]. According to the literature, the resultant velocity of the shot, the angle of release, and the height of the center of the shot during release were the most important factors influencing the distance of the throw. Unfortunately, the last indicator is dependent on human anthropometrics [7,8,9], so it cannot be changed in the training process. Indicators like the height of the athlete’s center of gravity and the height of the center of the shot had low variability (below 5%), according to RE, regardless of the sports level and the technique. The analysis of release indicators for different groups of athletes during comparable competition conditions (group B from our study and comparative data from the study of Gutiérrez-Davila et al. [17]) proved that the height of the shot was relatively constant and had low variability. Gutiérrez-Davila et al. [17] showed that the angle of release in athletes using rotational techniques varied more than in athletes using the glide technique. It was probably the effect of the greater deviation during the rotation phase and lower stability of the athlete. Greater RE of selected indicators in group B from our study in relation to comparative data from the study of Gutiérrez-Davila et al. [17] (all throws were over 20 m) was probably caused by lower levels of technical skills manifested by the former group.

In general, in our research, low RE characterized RLS indicators and the indicators of the path of athletes’ center of gravity and the center of the shot. Moreover, lower RE was found in the trials of higher-level athletes (group A). The greatest differences between groups A and B were found in the rotational technique for S-H, Vy_CG and Vx_CG. The greatest differences in the glide technique were also observed for S-H, Vy_CG and Vy_S. It was only in the case of Vx_CG that general average RE (in the rotational and glide techniques) in group A was found to be greater than in group B. The average RE for the 8 finalists from our study group A–international level (5 athletes using the rotational technique and 3 athletes using the glide technique) compared to group in the study of Shaa [15], also with 8 athletes (5 using the rotational technique and 3 using the glide technique) showed similar low variability (according to RE) for the angle of release and the distance of the throw (in all cases below 10%).

Taking into account all the analyzed athletes, without the division into sports levels and techniques used, significant (at *p* < 0.05) linear dependence was found between the longest trials and the relative errors of release indicators for the longest distance of each athlete. It was observed that while the distance was increasing, the value of RE was decreasing linearly for 4 release indicators (the angle of the right and left knee, resultant velocity of the athlete’s center of gravity and horizontal velocity of the shot) in the glide technique in group A, for 2 indicators (horizontal velocity of the athlete’s center of gravity and the shot) in the glide technique in group B, and for 3 indicators (the angle of the right knee, horizontal and resultant velocity of the shot) in the rotational technique in group B. The lowest average values of the relative error for the longest trials were noted in 16 cases in the glide technique in group A, in 6 cases in the glide technique in group B, in 5 cases in the rotational technique in group A and in 7 cases in the rotational technique in group B. The longest trial in the rotational technique, without the division into sports level and in relation to the other distances, had the lowest average value of the relative error for 3 indicators (shoulder-hip separation, vertical distance of the athlete’s center of gravity and the resultant distance of the center of the shot). In the glide technique, without the division into sports level, the longest trial had the lowest average value of the relative error for 7 cases (the angle of the right elbow, shoulder-hip separation, the height of the shot and the athlete’s center of gravity, vertical velocity of the shot, the distance of the shot in the vertical and lateral direction). The results of this study expanded the findings of previous optimization modeling attempts for elite men’s shot put [5,18,19,20,21,22]. Although the observations are not totally conclusive, they provide a large step forward towards a more complete optimization model for the task. 

## 5. Conclusions

The results of this study suggest that selected parameters play an important role in the performance of elite men’s shot put. Among all the analyzed indicators, the RLS factors of the shot (γ, H, Vx, Vy, Vr) had the lowest mean values of RE, especially in the elite group. A significant influence of the resultant shot velocity on the distance was confirmed. Also, the right knee joint angle and the left hip joint angle had a significant effect on the distance of the throw. The greatest differences between the elite and sub-elite group were found for S-H and Vy_CG. Further research on the topic is necessary to better understand all of the relationships both between these variables and with performance. The observations of this study provide useful information for the technical development of male throwers and may provide an insight into new training methods that should mainly be focused on the power development associated with the mode of the shot put technique. The research results show that greater repeatability of the technique (lower RE) has a significant impact on the length of the shot put. Along with the decrease in RE, the distance of the shot put increased. The relationship between the sports level and greater repeatability confirms the importance of the technique automatism and stabilization of the technique in sport.

## Figures and Tables

**Table 1:** The kinematic parameters selected for the analysis.

Symbol	Description
γ	RLS angle
H	height of RLS
h	height of the center of the body during RLS
D	distance of the throw
Vr	resultant velocity of the athlete’s center of gravity (CG) and the center of the shot (S)
Vx	horizontal velocity of the athlete’s center of gravity (CG) and the center of the shot (S)
Vy	vertical velocity of the athlete’s center of gravity (CG) and the center of the shot (S)
S-H	shoulder-hip separation during RLS
β_r	rear (right) knee angle
β_l	front (left) knee angle
φ_r	shoulder angle of the right (throwing) arm
δ_r	elbow angle of the right (throwing) arm
λ_r	right hip angle
λ_l	left hip angle
x_CG	distance in horizontal directions of the athlete’s center of gravity
y_CG	distance in vertical directions of the athlete’s center of gravity
z_CG	distance in lateral directions of the athlete’s center of gravity
R_CG	resultant distance of the athlete’s center of gravity
x_S	distance in horizontal directions of the shot
y_S	distance in vertical directions of the shot
z_S	distance in lateral directions of the shot
R_S	resultant distance of the shot

**Table 2:** Average (±SD) kinematic parameters of the athlete and the shot during release depending on the group (A and B) and the technique (R–rotational technique, and G–glide technique).

	A		B	
	R	G	R	G
β_r (°)	148.31 ± 10.16	141.74 ± 11.06	150.00 ± 16.14	133.04 ± 34.56
β_l (°)	168.31 ± 6.18	172.83 ± 3.31	170.25 ± 4.71	175.92 ± 3.03
φ_r (°)	122.54 ± 4.43	131.80 ± 3.83	121.03 ± 7.49	121.83 ± 14.01
δ_r (°)	162.01 ± 9.63	164.09 ± 7.82	147.55 ± 15.78	137.06 ± 14.61
λ_r (°)	167.89 ± 5.90	171.46 ± 3.71	162.76 ± 7.18	169.05 ± 5.54
λ_l (°)	156.11 ± 7.30	141.95 ± 5.23	147.77 ± 4.26	139.98 ± 8.97
S-H (°)	23.99 ± 11.00	30.56 ± 6.28	31.95 ± 12.48	22.75 ± 6.59
VxCG (m/s)	0.55 ± 0.20	0.72 ± 0.32	0.60 ± 0.13	0.79 ± 0.22
VyCG (m/s)	0.99 ± 0.28	0.92 ± 0.17	1.31 ± 0.46	1.15 ± 0.28
VrCG (m/s)	1.22 ± 0.26	1.2 ± 0.25	1.52 ± 0.39	1.42 ± 0.31
h (m)	1.23 ± 0.07	1.23 ± 0.06	1.22 ± 0.08	1.24 ± 0.03
VxS (m/s)	10.23 ± 0.50	9.77 ± 0.68	9.49 ± 0.83	9.13 ± 0.90
VyS (m/s)	7.57 ± 0.33	7.86 ± 0.33	6.97 ± 0.60	7.55 ± 1.10
VrS (m/s)	12.76 ± 0.41	12.66 ± 0.46	11.83 ± 0.79	11.93 ± 0.87
H (m)	2.18 ± 0.11	2.27 ± 0.06	2.16 ± 0.16	2.27 ± 0.16
γ (°)	37.75 ± 3.32	39.40 ± 2.88	36.35 ± 2.86	39.57 ± 5.41
D (m)	18.95 ± 0.74	19.18 ± 1.02	18.08 ± 0.6	17.91 ± 0.5
x_CG (m)	1.56 ± 0.09	1.57 ± 0.11	1.57 ± 0.05	1.58 ± 0.41
y_CG (m)	0.71 ± 0.08	0.76 ± 0.15	0.72 ± 0.09	0.74 ± 0.20
z_CG (m)	0.72 ± 0.13	0.21 ± 0.04	0.79 ± 0.07	0.23 ± 0.08
R_CG (m)	2.04 ± 0.12	1.87 ± 0.18	2.15 ± 0.05	1.87 ± 0.49
x_S (m)	3.91 ± 0.39	3.71 ± 0.37	3.42 ± 0.41	3.20 ± 0.87
y_S (m)	2.37 ± 0.31	2.6 ± 0.25	2.07 ± 0.16	2.10 ± 0.64
z_S (m)	1.78 ± 0.22	0.71 ± 0.23	1.68 ± 0.24	0.47 ± 0.15
R_S (m)	5.42 ± 0.47	4.74 ± 0.49	4.88 ± 0.47	4.04 ± 1.09

**Table 3:** Mean percentage values of RE for the athlete and the shot (S) depending on the group (A and B) and the technique (R—rotational technique, and G—glide technique).

		CG	S			Athlete						
		h (%)	H (%)	D (%)	γ (%)	φ_r (%)	σ_r (%)	λ_p (%)	λ_l (%)	β_p (%)	β_l (%)	S-H [%)
A	R	5.0	1.2	1.9	3.2	2.8	3.6	2.1	1.4	1.8	1.2	20.5
	G	1.0	1.4	0.8	4.4	1.2	2.7	1.5	0.9	1.9	1.2	9.3
B	R	1.5	3.8	1.3	4.9	2.5	6.4	2.8	2.3	3.4	1.8	32.8
	G	1.9	4.3	2.3	9.9	8.7	9.8	2.5	2.4	2.3	1.1	19.4

**Table 4:** Mean percentage values of RE for the velocity and the distance (x, y, z and R) of the athlete’s center of gravity (CG) and the shot (S) depending on the group (A and B) and the technique (R–rotational technique, and G—glide technique).

		CG							S						
		x (%)	y (%)	z (%)	R (%)	Vx (%)	Vy (%)	Vr (%)	x (%)	y (%)	z (%)	R (%)	Vx (%)	Vy (%)	Vr (%)
A	R	1.9	2.3	4.6	1.8	21.8	9.0	6.2	5.7	6.2	6.7	4.9	2.2	2.3	1.4
	G	4.2	10.2	13.7	6.2	20.6	17.7	8.7	6.2	4.9	13.8	5.5	1.6	3.6	1.3
B	R	1.8	3.4	6.0	1.8	17.6	20.0	13.1	5.0	5.7	3.5	4.2	5.9	5.9	5.1
	G	2.1	3.9	13.2	2.0	18.4	18.7	15.6	7.2	11.7	9.6	6.8	5.1	10.4	5.1

**Table 5:** The values of the statistically significant correlation coefficients between the RE level of kinematic parameters and the distance of the throw. Statistically insignificant correlations between the RE level of kinematic parameters and the distance of the throw are not represented.

TECHNIQUE	R + G	R	G	R + G	R + G	R	R	G	G
GROUP	A + B	A + B	A + B	A	B	A	B	A	B
Parameters of the athlete									
β_r	−0.250	**−0.380**	−0.156	0.106	**−0.551**	−0.125	**−0.623**	0.346	**−0.626**
β_l	**−0.288**	−0.291	−0.303	−0.183	−0.374	0.040	**−0.692**	−0.305	−0.428
σ_r	**−0.304**	−0.210	−0.322	0.136	−0.027	0.049	0.099	0.232	−0.062
λ_l	**−0.270**	−0.120	−0.369	0.288	−0.277	0.378	−0.441	0.281	−0.150
S-H	−0.217	**−0.411**	−0.199	−0.077	−0.134	−0.092	−0.413	−0.006	−0.117
VxCG	−0.228	−0.161	−0.207	−0.271	−0.339	−0.031	−0.473	**−0.591**	−0.522
VyCG	−0.153	0.036	−0.298	−0.021	0.033	0.435	−0.044	**−0.718**	0.046
Parameters of the shot									
VxS	**−0.460**	**−0.483**	**−0.475**	−0.246	**−0.441**	−0.229	−0.575	−0.259	−0.406
VrS	**−0.531**	**−0.653**	−0.358	−0.225	−0.376	**−0.656**	−0.325	0.398	−0.130
γ	**−0.305**	**−0.368**	−0.117	−0.017	−0.139	−0.307	−0.266	0.421	0.237
x_S	−0.153	−0.129	−0.056	−0.064	0.355	0.216	0.239	**−0.684**	0.427

The TECHNIQUE factor (R—rotational, G—glide, R + G—regardless of the technique) and the GROUP factor (A—international level, B—national level, A + B–regardless of the sports level) were taken into account. Significant (*p* < 0.05) correlations are in bold.

## Data Availability

Data are available upon reasonable request from the corresponding author.
